# Combinatorial antitumor effect of HDACs and the PI3K-Akt-mTOR pathway inhibition in a Pten deficient model of prostate cancer

**DOI:** 10.18632/oncotarget.1314

**Published:** 2013-09-27

**Authors:** Leigh Ellis, ShengYu Ku, Swathi Ramakrishnan, Elena Lasorsa, Gizzou Azabdaftari, Alejandro Godoy, Roberto Pili

**Affiliations:** ^1^ Genitourinary Program, Roswell Park Cancer Institute, Buffalo NY; ^2^ Department of Pharmacology and Therapeutics, Roswell Park Cancer Institute, Buffalo NY; ^3^ Department of Cancer Prevention and Pathology, Roswell Park Cancer Institute, Buffalo NY; ^4^ Department of Pathology, Roswell Park Cancer Institute, Buffalo NY; ^5^ Department of Urology, Roswell Park Cancer Institute, Buffalo NY; ^6^ Department of Medicine. Roswell Park Cancer Institute, Buffalo NY; ^7^ Department of Physiology, Pontificia Universidad Católica de Chile, Santiago, Chile.

**Keywords:** Prostate cancer, EBZ235, panobinostat, DNA damage, ATM

## Abstract

Increased expression of histone deacetylases (HDACs) and activation of the PI3K-Akt-mTORC1 pathway are common aberrations in prostate cancer (PCa). For this reason, inhibition of such targets is an exciting avenue for the development of novel therapeutic strategies to treat patients with advanced PCa. Previous reports demonstrated that HDAC inhibitors (HDACi) increases DNA damage and induce greater apoptosis in PCa cell lines that express androgen receptor (AR). In this study we utilized the AR negative PCa cell line and observed that re-expression of AR (PC3-AR) results in greater levels of apoptosis when treated with the pan-DACi, panobinostat (PAN). PAN mediated apoptosis in PC3 and PC3-AR cells was associated with increased levels of double strand DNA breaks, indicated by p-ɣH2AX. Further, PAN treatment in PC3-AR cells resulted in moderate attenuation of the ATM-Akt-ERK DNA damage response pathway. For this reason, we combined PAN with the dual PI3K-mTOR inhibitor, BEZ235. Combination of PAN with BEZ235 resulted in significant attenuation of the DNA damage repair protein ATM and significantly increased anti-tumor activity compared to each single treatment. Overall, superior anti-tumor activity with combination of PAN with BEZ235 was independent of AR status. These findings suggest that this therapeutic strategy should be further developed in clinical trials.

## INTRODUCTION

Castrate resistant prostate cancer (CRPC) represents an aggressive and incurable phenotype of prostate cancer. Until recently, patients who progressed after receiving androgen deprivation therapy (ADT) had minimal second line therapeutic options available. Within the last 2 years a dramatic shift in the therapeutic landscape for CRPC has emerged, resulting in the FDA approval of 5 novel agents [1-5]. Although these therapies have resulted in increased survival benefits for CRPC patients, sustainable suppression of CRPC growth still remains a primary challenge and novel treatment strategies are still required.

Histone deactelyases (HDACs) are demonstrated to be over-expressed in multiple cancers, including prostate cancer [6-8]. HDAC inhibitors (HDACi) are a heterogeneous group of epigenetic therapies which alter histone and non-histone protein function [9]. HDACi have demonstrated preclinical and clinical efficacy as monotherapy [10, 11] and also combination therapy [12, 13]. HDACi have a diverse range of anticancer activity including induction of tumor cell apoptosis [10], suppression of angiogenesis [14] and DNA damage repair (DDR) [15] and induction of DNA damage by double strand DNA breaks (DSB) [16].

The PI3K-Akt-mTORC1 signaling axis is often constitutively activated in prostate cancer, primarily due to loss of expression/function of the tumor suppressor gene *Pten* [17]. Recently, Akt signaling has been implicated in modulating DNA damage responses and genomic instability [18]. Akt activation in response to DNA damage has been shown to be induced by the PI3-like kinase kinases (PI3KK) ataxia telangiectasia-mutated (ATM), ataxia telangiectasia and Rad3-related (ATR) and DNA-dependent protein kinase (DNA-PK), all of which are activated by DNA damage [19]. Activation of Akt by these PIKKs in response to DNA damage is proposed to initiate prosurvival signaling via Akt mediated cell cycle arrest and anti-apoptotic mechanisms [20-23].

Selective mTORC1 inhibitors including rapamycin and its analogs have been investigated for the treatment of prostate cancer, though with limited success. This failure is thought to be due to the reciprocal feedback loop and activation of rapamycin-insensitive (mTORC2) mTOR complex leading to Akt activation [24-26] and increased activity of AR [12]. Accordingly, inhibitors that target PI3K and both rapamycin-sensitive (mTORC1) and –insensitive (mTORC2) have been developed [27]. These inhibitors demonstrate successful abrogation of the feedback activation of Akt.

BEZ235 (Novartis) is an orally available PI3K/mTOR inhibitor [27] that demonstrated efficacy in inhibiting tumor growth in preclinical mouse models [28-35]. Treatment with BEZ235 of multiple tumor types has resulted in potent inhibition of Akt, mTORC1 and mTORC2 activity. However, it has been recently demonstrated that BEZ235 inhibits the DDR proteins DNA-PK and ATM [36], both which share high homology of their catalytic domain with PI3K [37].

The PC3 androgen independent prostate cancer cell line, which is devoid of AR, Pten and p53 expression is shown to be resistant to HDACi mediated apoptosis. In our study, we have utilized a PC3 cell line that expresses AR (PC3-AR) to demonstrate that treatment of PC3-AR cells with the pan-DACi panobinostat induces apoptosis. Panobinostat treatment resulted in comparable reduction of activated Akt and ATM in PC3 cells and PC3-AR cells. However, we observed a dramatic increase of double strand breaks in PC3-AR cells compared to PC3 cells. Further, we determine that *in vivo*, combination of panobinostat with BEZ235 resulted in greater anti-tumor activity compared to single treatment against PC3 and PC3-AR tumors. Anti-tumor activity mediated by panobinostat/BEZ235 combination was associated with sustained or increased levels of DNA damage with concurrent reduction of the DDR protein ATM. These data suggest that combination of panobinostat and BEZ235 results in superior therapeutic efficacy that is independent of AR and p53 status. We conclude that combined HDAC and DDR inhibition represents a novel therapeutic strategy for advanced and/or CRPC.

## RESULTS

**Figure 1:**
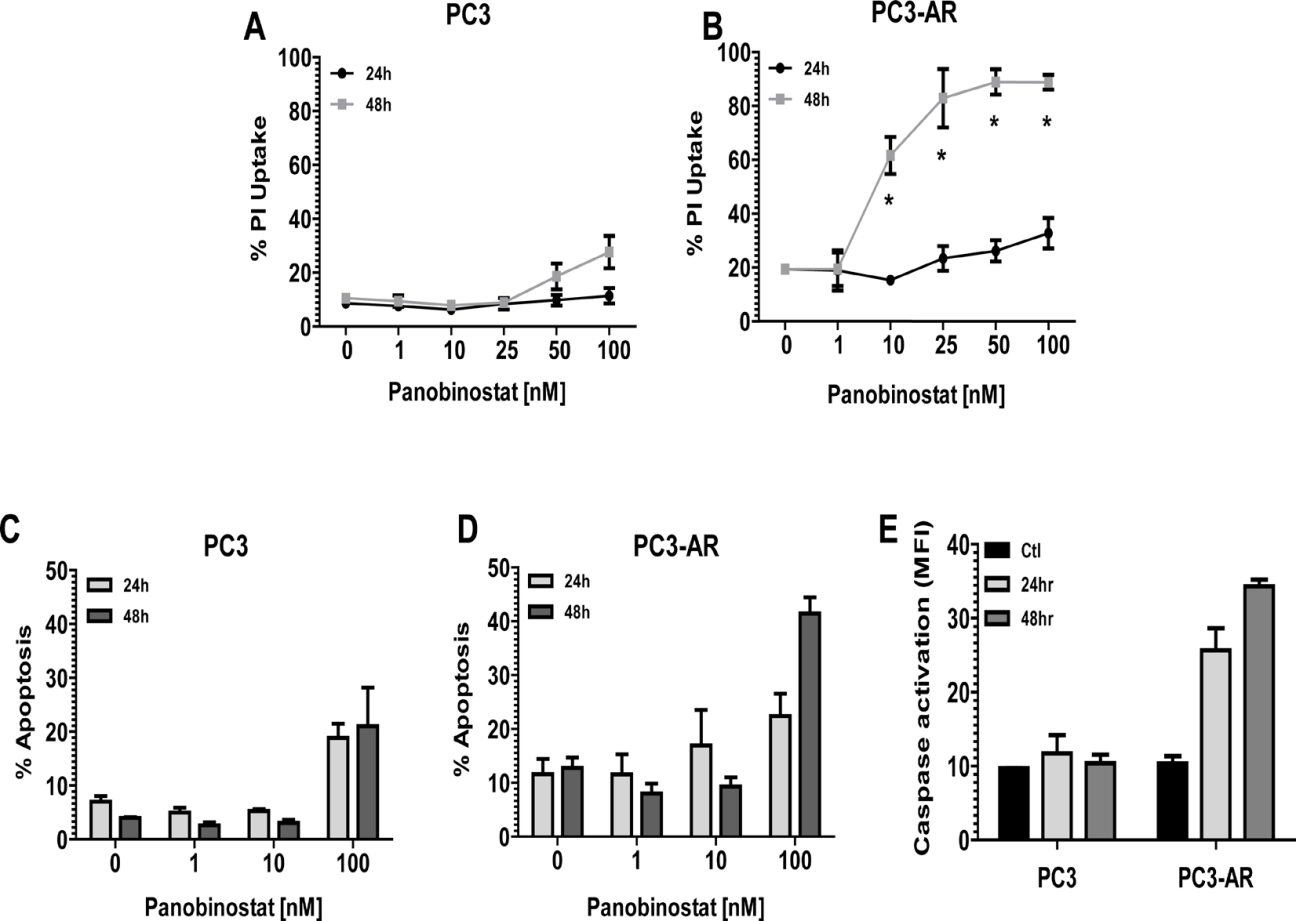
(A-B) PC3 cells and PC3-AR cells were treated with increasing concentrations of panobinostat (PAN) for 24 and 48 hours Cells were trypsinized and washed in 1x PBS and incubated with propidium iodide (PI). Percentages of PI positive cells were determined by flow cytometry. (C-D) PC3 cells and PC3-AR cells were treated with increasing concentrations of panobinostat (PAN) for 24 and 48 hours. Cells were trypsinized and washed in 1x PBS and incubated with annexin V. Percentages of annexin V positive cells were determined by flow cytometry. (E) PC3 cells and PC3-AR cells were treated with 100nM panobinostat (PAN) for 24 and 48 hours. Cells were trypsinized and washed in 1x PBS and incubated with fluoroisothiocyanate (FITC)-VAD to detect activation of caspases 3, 7 and 9. Percentage of positive cells for activated caspases was determined by flow cytometry. All data represent the mean of 3 independent experiments ±SE. * p < 0.05 (two-tailed t-test).

**Figure 2:**
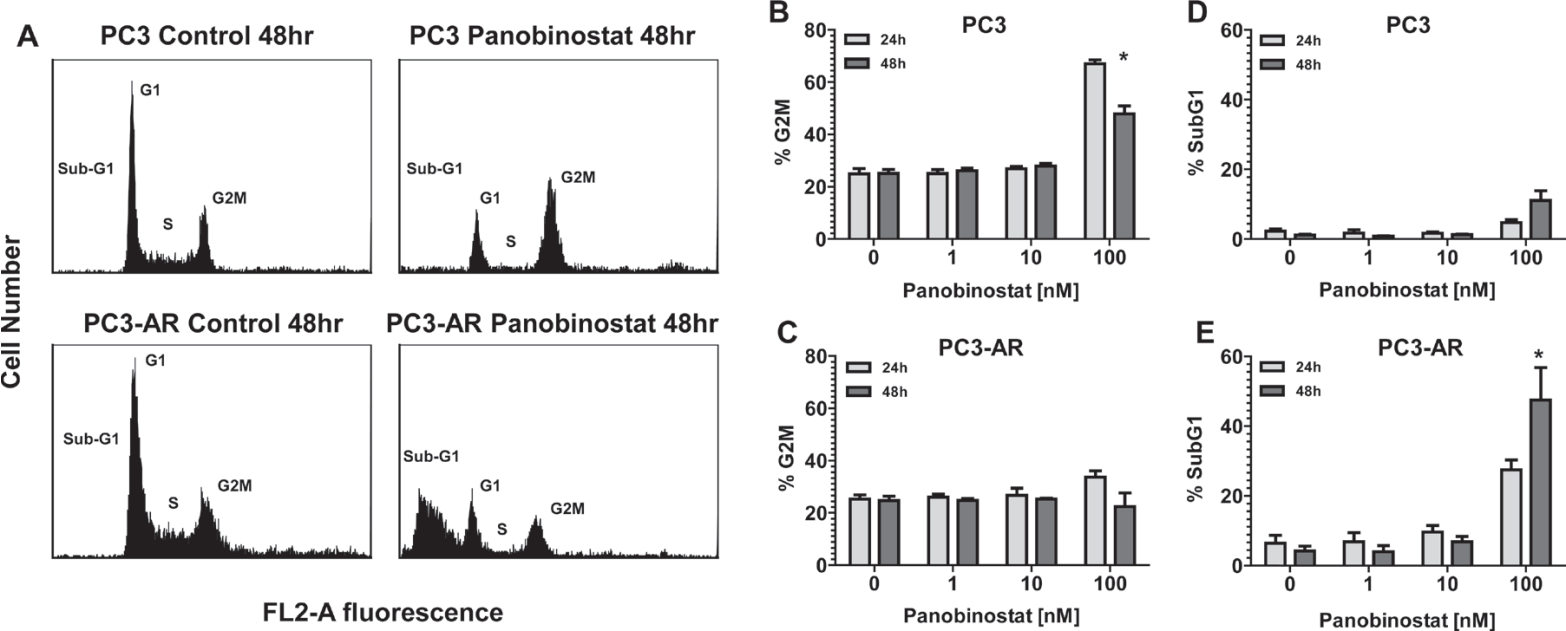
PC3 and PC3-AR cells were treated with increasing concentrations of panobinostat for 24 and 48 hours Cells were trypsinized and washed in 1x PBS before being fixed in 70% ethanol and stained with PI. Cell cycle distribution was assessed by flow cytometry. (A) Representative histograms of cell cycle profile of PC3 and PC3-AR cells treated with 100nM panobinostat for 48 hours. (B-C) Quantitated measurements of G_2_M cell cycle distribution and (D-E) subG_1_ cell cycle distribution for PC3 and PC3-AR cells following panobinostat treatment. Columns represent mean of 3 independent experiments ±SE.* p < 0.05 (two-tailed t-test).

Androgen receptor expression sensitizes PC3 cells to HDACi mediated apoptosis. PC3 and PC3-AR cells were exposed for 24 and 48 hours to increasing concentrations of the HDACi panobinostat (PAN) and plasma membrane integrity assessed by uptake of propidium iodide (PI). Initial confirmation of the functional status of AR expressed in PC3-AR cells is shown in supplement Fig. 1. As shown in Fig. 1A, only 100nM PAN slightly increased killing of PC3 cells. In contrast, low-nanomolar concentrations of PAN were sufficient to kill PC3-AR cells in a time dependent manner (Fig. 1B). We next assessed the biochemical changes that occurred after treatment of PC3 and PC3-AR cells with PAN. Only PC3-AR cells treated with 100nM PAN displayed hallmark features of apoptosis compared to the same treatment of PC3 cells. These included increased cell surface exposure of phosphatidyserine (Fig. 1C and 1D) and caspase activation (Fig. 1E). Further, treatment of PC3 and PC3-AR cells with 100nM PAN resulted in accumulation of subG_1_ population in PC3-AR cells indicating DNA fragmentation, whereas the same treatment resulted in cell cycle arrest of PC3 cells in the G_2_M phase of the cell cycle (Fig. 2).

### Panobinostat treatment increases DNA damage and suppresses expression of activated ATM, Akt and Erk1/2 protein

**Figure 3:**
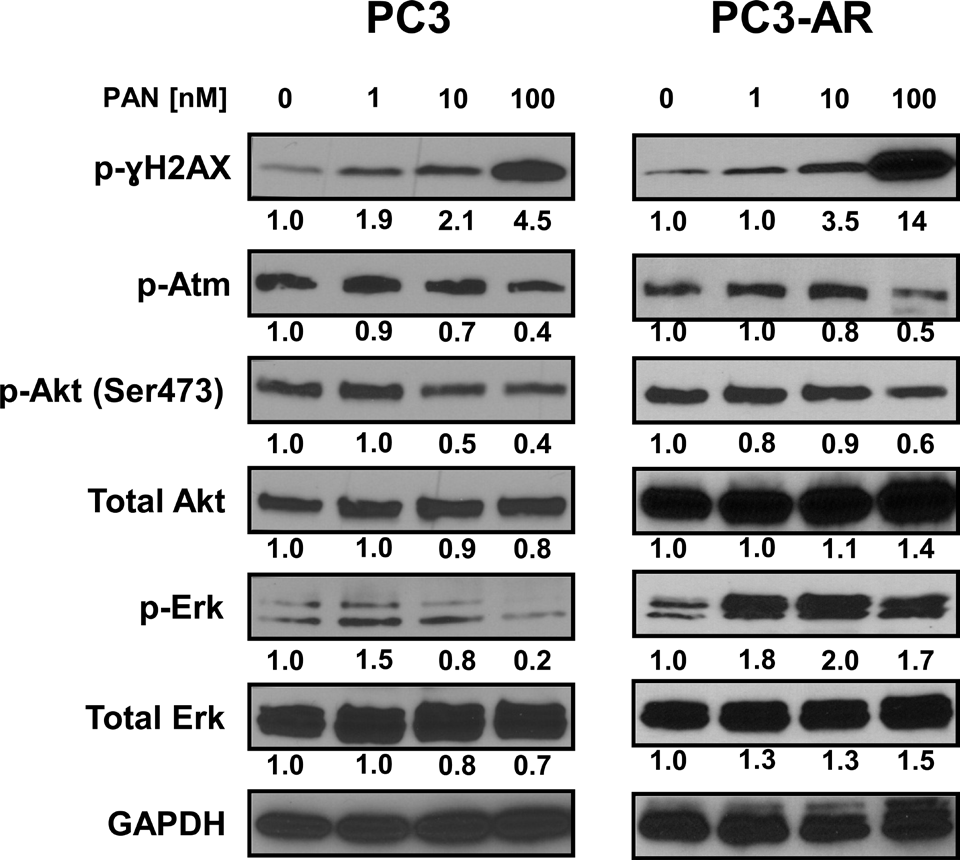
PC3 and PC3-AR cells were treated with increasing concentrations of panobinostat for 24 hours Following treatment, total cell lysates were prepared and immunoblot analysis were performed for p-ɣH2AX (ser139), p-Atm (Ser1981), p-Akt (Ser473), Akt, p-Erk1/2 and Erk1/2. The expression of GAPDH in lysates served as the loading control. The numbers below the bands represent densitometry performed by image j analysis on representative immunoblots relative to the control cells.

It had been previously demonstrated that HDACi possess the ability to induce DNA damage and apoptosis [16]. Further, PC3 cells are devoid of Pten, leading to constitutive activation of Akt, and it is documented that part of the DNA damage response pathway can be mediated through ATM-Akt-Erk1/2 signaling [38]. We therefore examined the sensitivity of this pathway in response to PAN. PC3 and PC3-AR cells treated with increasing concentrations for 24 hours showed a dose dependent increase in DNA damage, as indicated by protein levels of p-ɣH2AX (Fig. 3). Increases in DNA damage were most noticeable at a PAN concentration of 100nM (PC3; 4.5 fold increase, PC3-AR; 14 fold increase), which correlated with the PAN mediated cell killing in both cell lines. Further examination revealed that 100nM PAN resulted in similar attenuation of p-ATM and p-Akt in both cell lines (Fig. 3). PAN treatment (100nM) in PC3 cells decreased p-Erk1/2 levels, though at the same concentration, p-Erk1/2 levels were increased in PC3-AR cells (Fig. 3). Taken together, these results suggest that the greater accumulation of DNA damage in PC3-AR cells following PAN treatment may partially explain the ability of PAN to induce apoptosis in AR expressing PC3 cells.

**Figure 4:**
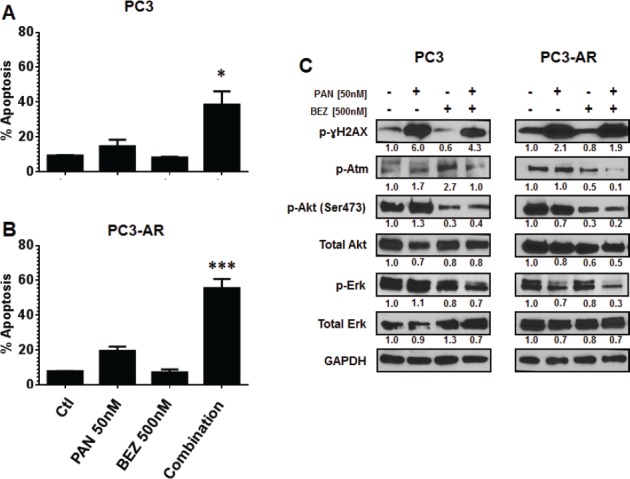
(A-B) PC3 cells and PC3-AR cells were treated with indicated concentrations of panobinostat and/or BEZ235 for 48 hours Cells were trypsinized and washed in 1x PBS and stained with annexin V dye. Annexin V positive cells were assessed by flow cytometry. Columns represent mean of three independent experiments ±SE. * indicates p = 0.01 for combination treatment compared to single treatments by one-way ANOVA. *** indicates p = 0.0002 for combination treatment compared to single treatments by one-way ANOVA. (C) PC3 and PC3-AR cells were treated with indicated concentrations of panobinostat and/or BEZ235 for 24 hours. Following treatment, total cell lysates were prepared and immunoblot analysis was performed for p-ɣH2AX (ser139), p-Atm (Ser1981), p-Akt (Ser473), Akt, p-Erk1/2 and Erk1/2. The expression of GAPDH in lysates served as the loading control. The numbers below the bands represent densitometry performed by image j analysis on representative immunoblots relative to the control cells.

Combination of panobinostat and BEZ235 results in increased apoptosis in PC3 and PC3-AR cells. BEZ235 has recently been reported to inhibit the DNA damage response through attenuation of DNA-PKCs and ATM [35, 36]. Investigation of these two agents in combination treatment of PC3 and PC3-AR cells resulted in significant increase in apoptosis compared to each single treatment (Fig. 4A and Fig. 4B; PC3, p=0.01 combination verse single treatment and PC-AR, p=0.0002 combination verse single treatment as determined by one-way ANOVA). Initial immunoblot performed demonstrated that BEZ235 induced potent inhibition of the downstream targets of mTORC1, p-4EBP1 and p-S6K (supplement Fig. 3A). Next, we examined if increased apoptosis following combination treatment was associated with increased DNA damage and loss of activated ATM, Akt and Erk1/2. PAN treatment of either cell line increased levels of p-ɣH2AX as expected and was maintained in combination treatment (Fig. 4C). Combination treatment resulted in similar loss of p-Akt compared to BEZ235 in PC3 and PC3-AR cell lines. Phospho-Erk1/2 was not greatly reduced in PC3 cells by any treatment in contrast to combination treatment of PC3-AR (Fig. 4C). Interestingly, levels of p-ATM were initially increased by either PAN or BEZ235 treatment in PC3 cells, but combination treatment maintained p-ATM levels similar to that in untreated cells. Conversely, PAN treatment of PC3-AR cells did not affect p-ATM levels, and BEZ235 treatment resulted in reduction of p-ATM levels. This reduction of p-ATM was enhanced by combination treatment of PC3-AR cells (Fig. 4C). Collectively, this data demonstrates the potential of PAN combined with BEZ235 to maintain increased levels of DNA damage while attenuating the DNA damage response mediated through ATM-Akt-Erk1/2 signaling.

**Figure 5:**
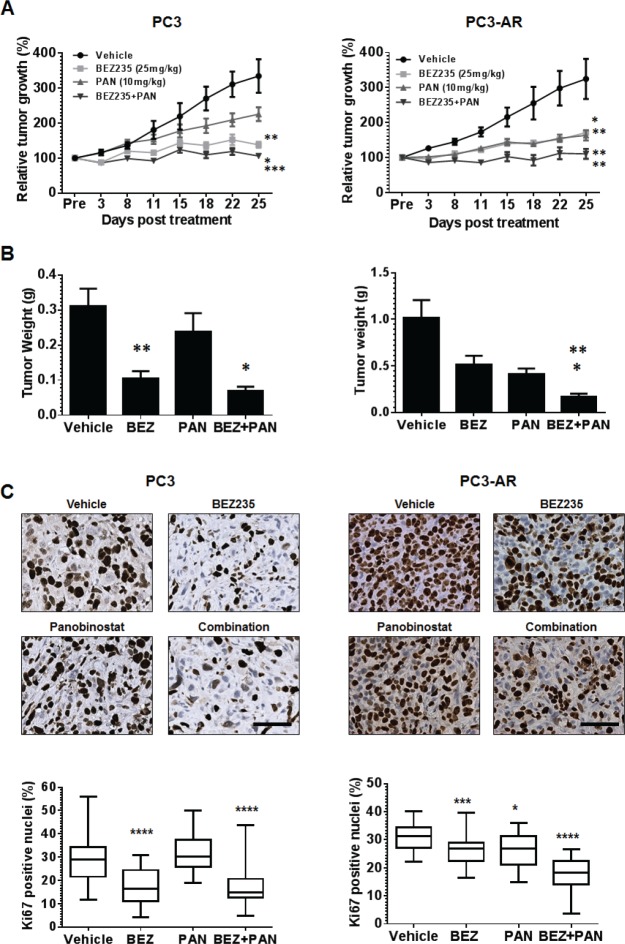
(A) PC3 or PC3-AR cells (5×10^6^) were injected subcutaneously in the flank of intact SCID male mice Treatment was initiated when tumors measured approximately 50mm^2^ (L x W). Mice were treated with vehicle (NMP:PEG300 1:9, 5d on 2d off, oral gavage, PC3 *n=8*, PC3-AR *n=5*), BEZ235 (25mg/kg, 5d on 2d off, oral gavage x5 weekly, PC3 *n=8*, PC3-AR *n=6*), panobinostat (PAN) (10mg/kg, Mon-Wed-Fri, i.p., PC3 *n=8*, PC3-AR *n=7*), or combination (PC3 *n=8*, PC3-AR *n=6*). Tumor size was monitored by serial caliper measurements biweekly. Tumor size was calculated by L x W. Each treatment group was normalized to the pretreatment measurements and converted to percent tumor growth. Each point represents mean tumor size ±SE. For PC3 tumors, * indicates significant difference between BEZ235 and combination (p=0.02), ** p=0.002 (BEZ235 vs. vehicle) and *** p=0.0002 (combination vs. PAN) as determined by two-way ANOVA. For PC3-AR tumors, * indicates significant difference vehicle and BEZ235 (p=0.01), ** p=0.005 (PAN v.s vehicle), ** p=0.007 (BEZ235 vs. combination and ** p=0.007 (PAN vs. combination) as determined by two-way ANOVA. (B) endpoint tumor weights; results for each treatment group represent mean ±SE. For PC3 tumors, ** p=0.001 (vehicle vs. BEZ235), * p=0.01 (BEZ235 vs. combination). For PC3-AR tumors, * p=0.03 (PAN vs. combination), ** p=0.003 (BEZ235 vs. combination), as determined by two-tailed, paired t-test. (C) Example photomicrographs of Ki67 immunostained PC3 and PC3-AR tumor tissue. For PC3 tumors, **** indicates significant difference between vehicle and BEZ235 (p<0.0001), **** p<0.0001 (combination vs. BEZ235). For PC3-AR tumors, * p=0.01 (PAN verse vehicle), *** p=0.0007 (BEZ235 vs. vehicle), **** p<0.0001 (combination vs. PAN and BEZ235), as determined by two-tailed, paired t-test.

BEZ235 and PAN combined treatment of PC3 and PC3-AR produces greater antitumor activity. The antitumor activity of BEZ235 and PAN was investigated *in vivo* by treating male SCID mice bearing PC3 or PC3-AR subcutaneous tumors. Tumor bearing mice were treated with vehicle (*N*-Methyl-2-pyrrolidone (NMP)/PEG300, 1:9 v/v), PAN (10mg/kg i.p.: Mon-Wed-Fri), BEZ235 (25mg/kg i.p.: Mon-Fri) or combination. Treatment of PC3 or PC3-AR tumor bearing mice did not result in significant toxicity by any treatment (Supplement Fig. 2A and 2B). Pathological assessment of hematoxylin and eosin staining revealed that treatment of PC3 or PC3-AR tumors did not result in any distinctive morphological changes (data not shown). As seen in Fig. 5A BEZ235 single treatment resulted in significant anti-tumor activity towards PC3 and PC3-AR tumors (PC3, p=0.003; PC3-AR, p=0.01). Single treatment with PAN inhibited PC3 tumor growth modestly but did significantly inhibit PC3-AR tumors when compared to vehicle treated mice (PC3, p=0.1; PC3-AR, p=0.005). Interestingly, combination therapy demonstrated significant therapy when compared to single treatment of PC3 tumors (p=0.02, combination vs. BEZ235; p=0.0002, combination verse PAN) and PC3-AR tumors (p=0.007, combination vs. BEZ235; p=0.008, combination vs. PAN). These data was also supported by the analysis of end-point PC3 and PC3-AR tumor weights (Fig. 5B and supplement Fig. 2C).

Immunohistochemical analysis for p-S6K replicated *in vitro* results and demonstrated that BEZ235 was a potent inhibitor of downstream mTORC1 signaling, that was further enhanced by combination treatment with PAN (supplement Fig. 3B). Immunostaining for the proliferation marker Ki67 revealed that, compared to vehicle treated tumors, BEZ235 single treatment resulted in significant reduction of PC3 and PC3-AR tumor proliferation (PC3, p<0.0001; PC3-AR, p=0.0007). Further, PAN single treatment did not significantly reduce PC3 tumor proliferation but did significantly inhibit PC3-AR tumor proliferation (PC3, p=0.6; PC3-AR, p=0.01). BEZ235/PAN combination treatment of PC3 tumors did impair tumor proliferation further compared to BEZ235 single treatment, though did significantly reduce PC3 tumor proliferative capacity compared to PAN single treatment (p<0.0001). However, combination treatment of PC3-AR tumors resulted in dramatic reduction of tumor proliferation compared to each single treatment cohort (p<0.0001, combination vs. BEZ235; p<0.0001, combination vs. PAN). These results indicate that BEZ235/PAN combination treatment may provide a therapeutic strategy for patients with advanced prostate cancer independent of tumor AR status.

**Figure 6:**
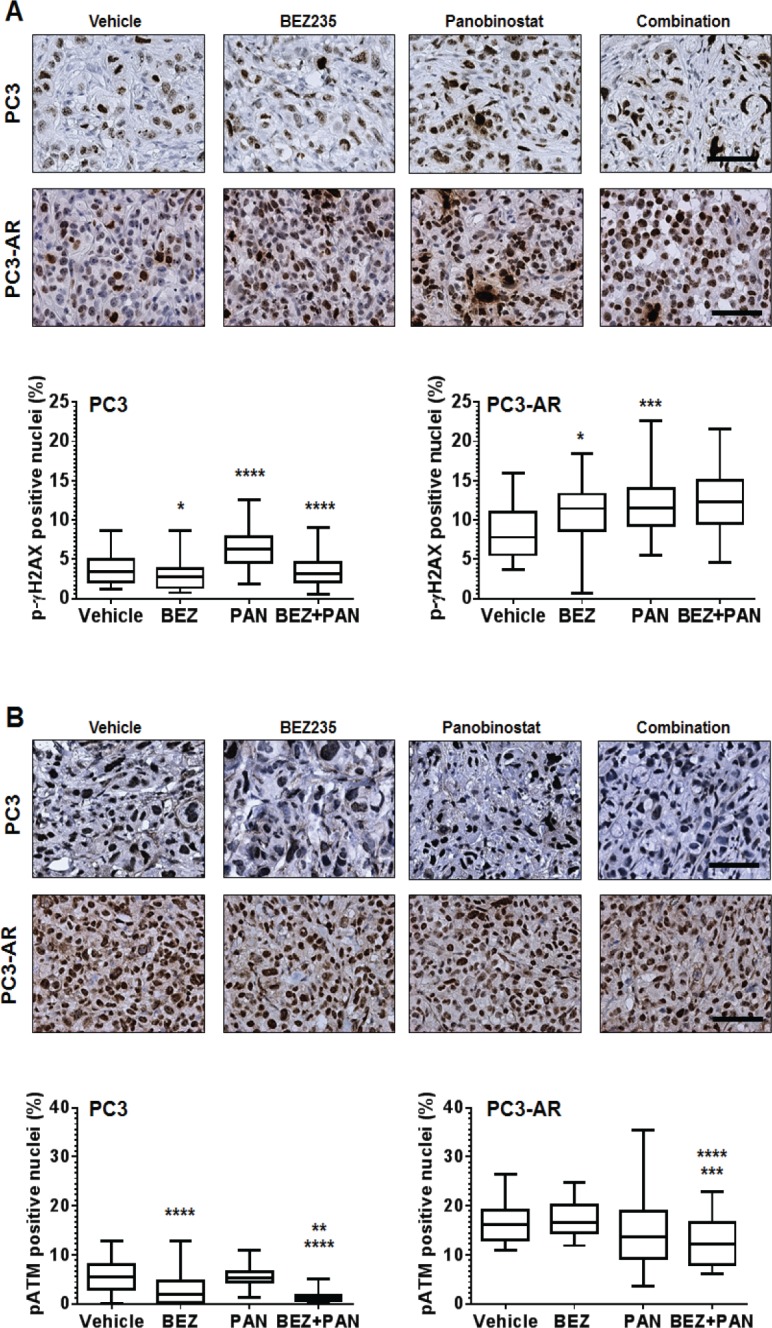
(A) Representative photomicrographs of p-ɣH2AX immunostained PC3 and PC3-AR tumor tissue For PC3 tumors, * indicates significant difference between vehicle and BEZ235 (p=0.01), **** p<0.0001 (vehicle vs. PAN), **** p<0.0001 (combination vs. PAN). For PC3-AR tumors, * p=0.01 (BEZ235 vs. vehicle), *** p=0.0003 (PAN vs. vehicle), as determined by two-tailed, paired t-test. (B) Example photomicrographs of p-ATM immunostained PC3 and PC3-AR tumor tissue. For PC3 tumors, **** indicates significant difference between vehicle and BEZ235 (p<0.0001), ** p=0.006 (combination vs. BEZ235), **** p<0.0001 (combination vs. PAN). For PC3-AR tumors, **** p<0.0001 (combination vs. BEZ235), *** p=0.0001 (combination vs. PAN), as determined by two-tailed, paired t-test.

Combination with BEZ235 and PAN results in increased DNA damage and reduced ATM expression in PC3 and PC3-AR tumors. To assess the activity of BEZ235 and/or PAN on induction of DNA damage and inhibition of ATM expression *in vivo*, we used immunostaining to determine the expression of p-ɣH2AX and ATM. Fig. 6A shows that treatment of PC3 and PC3-AR tumors with BEZ235 resulted in tumor specific effects, where p-ɣH2AX levels were reduced in PC3 tumors (p=0.01) but increased in PC3-AR (p=0.01) tumors compared to vehicle treated tumors. As expected, PAN significantly increased p-ɣH2AX levels in both tumors (PC3, p<0.0001; PC3-AR, p=0.0003) compared to mice treated with vehicle. Combination treatment also resulted in tumor specific effects, as p-ɣH2AX was reduced to levels similar to vehicle treatment, though in PC3-AR tumors, p-ɣH2AX levels increased comparable to PAN single treated tumors.

Tissues were next assessed for p-ATM nuclear expression by immunostaining. PC3 tumors treated with BEZ235 alone resulted in a significant reduction of p-ATM compared to vehicle treated tumors (p<0.0001), whereas BEZ235 treatment of PC3-AR tumors did not significantly change levels of p-ATM. No significant changes were observed in either PC3 or PC3-AR tumors treated with PAN when compared to vehicle. However, compared to each single treatment, combination treatment in PC3 tumors significantly decreased p-ATM levels (p=0.006, combination verse BEZ235; p<0.0001, combination vs. PAN) and in PC3-AR tumors (p<0.0001, combination vs. BEZ235; p=0.0001, combination vs. PAN) (Fig. 6B). Collectively, these data indicate that greater anti-tumor activity resulting from combination of BEZ235 and PAN are associated with deregulation of the DNA damage-DNA damage repair equilibrium.

## DISCUSSION

Advances in drug development have resulted in exciting changes to the therapeutic landscape for advanced PCa. For several years, the primary treatment option of CRPC has been limited to docetaxel chemotherapy, which extends survival but for a limited time [39]. While the treatment of advanced PCa has been extremely challenging, novel therapeutic options have emerged. The second line cabazitaxel [1], the immunotherapy sipuluecel-T [2], the androgen synthesis inhibitor abiraterone acetate [3] the AR antagonist enzalutamide [4] and the alpha particle emitter radium-223 [5] have all received recent FDA approval for the treatment of advanced PCa. Although these new therapies have expanded the treatments options for patients, sustainable suppression of PCa growth still remains a primary challenge and new treatment strategies are still required.

Apoptosis induced by HDAC inhibition has been demonstrated to be dependent on AR expression [40]. Our study also demonstrates that PAN mediated apoptosis is AR dependent. PC3 cells response to PAN treatment was predominantly cell cycle arrest, whereas PC3-AR cells were significantly more sensitive to HDACi mediated apoptosis, as indicated by dramatic increases of cell membrane permeability, caspase activation and DNA fragmentation. The differential response to HDAC inhibition maybe explained by significant increase in DNA damage we observed in PC3-AR cells in response to HDACi. Transcription factors including AR are demonstrated to induce DNA damage via double strand breaks (DSBs) to mediate their transcriptional programs. These DSBs are recognized by DNA repair machinery such as poly (ADP-ribose) polymerase 1 (PARP1) and ATM. If not repaired properly, persistent DSBs can induce apoptosis [41]. We and others have shown that HDACi can mediate loss of AR transcriptional activity independent of AR degradation [12, 42], suggesting that cessation of AR transcription may also inhibit DNA damage repair, leading to greater cell catastrophe. This was further supported by the loss of activated ATM in response to HDACi, which would further abrogate DNA damage repair. ATM also mediates part of its DNA repair mechanism through the activation of Akt-Erk1/2 signaling [43]. Because the PC3 model used in our studies exhibits constitutively activated Akt via loss of the tumor suppressor gene *Pten*, we proposed that suppression of Akt-Erk1/2 signaling could be a critical axis underlying increased sensitivity to HDACi mediated DSBs and apoptosis. Previously, HDACi have been shown to attenuate activated levels of Akt and Erk1/2 [44, 45]. However, in our studies, comparable suppression of activated Akt and opposing effects of activated Erk1/2 in response to HDACi suggested that an imbalance in DSBs and ATM expression was critical for HDACI mediated apoptosis.

The ability of HDACi to create imbalance between DNA damage and DNA repair mechanisms was exciting, though the modest effect on Akt and Erk1/2 signaling suggested potential escape mechanisms which could be therapeutically exploited to enhance anti-tumor efficacy. Recently, the dual PI3K/mTOR inhibitor BEZ235 has been reported to exhibit potent *in vivo* anti-tumor activity via inhibition of Akt and both mTOR signaling complexes. Further, BEZ235 has also been shown to possess inhibitory action towards DNA damage response proteins DNA-PK and ATM in multiple tumor models [35, 36, 44, 46]. Here, combination of BEZ235 with PAN sustained DNA damage while abrogating activated ATM, Akt and Erk1/2 *in vitro*. Therefore, combining PAN with BEZ235 maintained the imbalance between DNA damage and DNA damage repair while simultaneously inhibiting Akt and Erk1/2 signaling, promoting greater accumulation of apoptotic cells.

The combination of PAN and BEZ235 *in vivo* showed significant attenuation of tumor growth in PC3 and PC3-AR tumors compared to single agent treatments, which was associated with pronounced loss of tumor proliferation. Combination treatment of PC3 tumors resulted in sustained DNA damage compared with vehicle treated tumors. However this level of DNA damage was less than that observed in PAN single treated tumors. This may be explained by the partial attenuation of DNA damage by BEZ235 single treatment. Moreover, PC3-AR tumors displayed increased DNA damage following combination treatment, comparable to PAN single treatment, and greater than vehicle and BEZ235 treated tumors. Most excitingly, in both tumor models, significant attenuation of the DNA damage response protein ATM occurred with combination therapy compared to single treatments. This is an important finding as it demonstrates the ability of this combination therapy to mediate therapeutic efficacy independent of tumor AR status.

The drug development of HDACi as single agents for the treatment of solid tumors, including prostate cancer, has been disappointing. Rational combination strategies that exploit the transcriptional and prostranslational modifications induced by HDACi should be clinically tested based on convincing preclinical data and robust co-clinical trials. Optimal dosing and schedule of administration should be based on the molecular mechanism targeted by the HDACi. Induction of apoptosis may require relatively high doses given in a pulsed fashion. Interestingly, the required *in vivo* dose capable of inducing a biological effect in the PC3 model was relatively low. Further molecular studies will help to dissect the genome and epigenome signature for this susceptibility and will provide the potential predictors of response in patients with PCa.

**Figure 7:**
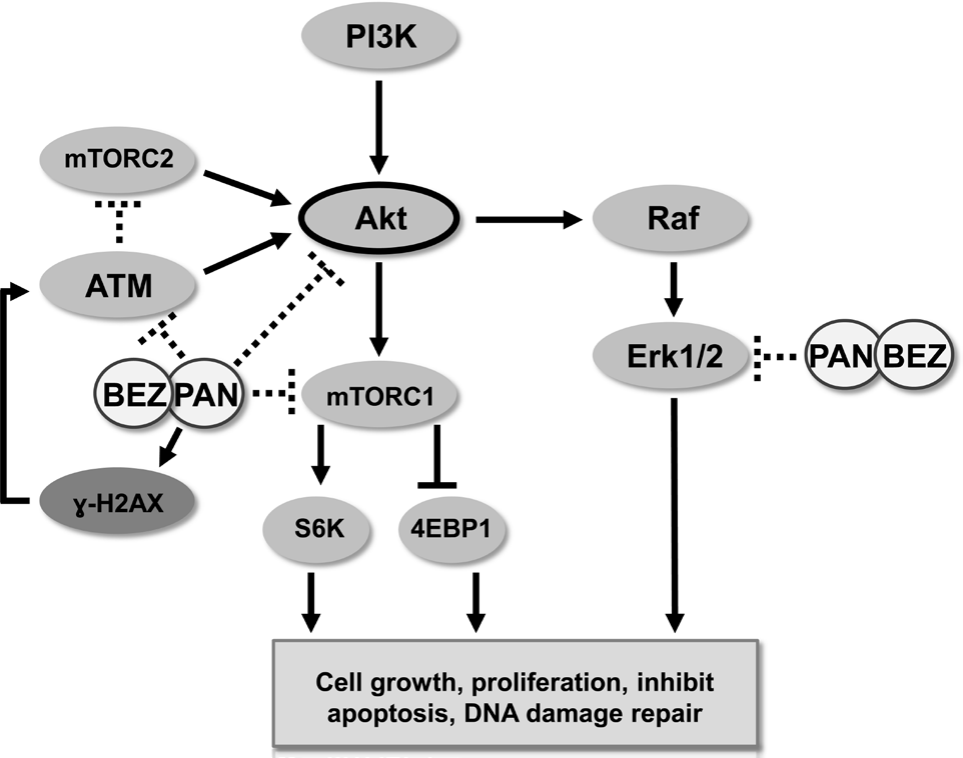
Schematic representation of activity of BEZ235/panobinostat combination against PC3 and PC3-AR tumor models Combination treatment of BEZ235 with panobinostat maintains/increases DNA double strand breaks as indicated by ɣ-H2AX. Further, combination treatment results in a superior suppression of the DNA damage response pathway mediated through ATM-Akt-Erk1/2. Overall, combination therapy creates an imbalance between DNA damage and DNA damage response generating greater therapeutic efficacy.

In conclusion, targeting advanced prostate cancer largely involves inhibitors towards androgen synthesis and androgen receptor transactivation. Together, our data provides rationale for the clinical translation of PAN combined with BEZ235 for treatment of advanced prostate cancer, independent of AR status. While targeting the DNA damage/DNA repair axis has been a goal of chemotherapy intervention, toxicity has made this strategy difficult to manage clinically. Both PAN and BEZ235 have returned favorable toxicity profiles in cancer patients and results from our study demonstrate that combination therapy sustains or increases intratumoral DNA damage while attenuating DNA damage repair (decreased ATM) and survival pathway (activated Akt and Erk1/2) signaling independent of AR status (Fig. 7). The optimal clinical setting to test this combination strategy remains to be determined but the molecular mechanism highlighted in this study suggests several potential rational combination strategies both pre and post docetaxel treatment. Overall, these data highlight the necessity of this therapeutic strategy to be further clinically developed to treat patients with advanced prostate cancer.

## MATERIALS AND METHODS

### Cell culture and Reagents

PC-3 cells were purchased from ATCC and PC3-AR [47] expressing cells were a kind gift from Dr. John Isaacs (John Hopkins University). All cell lines were cultured in RPMI-1640 medium supplemented with 10% fetal bovine serum and 1% penicillin/streptomycin at 37, 5% CO_2_. Primary antibodies used were GAPDH (#5174), p-Akt (Ser473, #4060), Akt (#9272), p-Erk1/2 (#4370), Erk1/2 (9102), p-ɣH2AX (Ser139, #9718), p-4EBP1 (#9451), 4EBP1 (9644), p-S6K (#2211), S6K (#2217) all from cell signaling, Ki67 (RM-9106, thermo scientific) and p-ATM (Ser1981; #2151-1, epitomics).

### Cell viability assay

One million cells (1×10^5^) were seeded in a 6-well plate (BD Bioscience) overnight to adhere. Cells were then incubated in the presence of indicated treatments for 24 and 48 hours. Following treatment, adherent and non-adherent cells were collected and washed in 1x PBS and stained by 500ng/ml of propidium iodide (PI) for 5 minutes. FACS Caliber cytometer was used to analyze PI uptake.

### Cell cycle analysis

One million cells (1×10^5^) were seeded in a 6-well plate (BD Bioscience) overnight to adhere and treated as indicated. Following treatments, adherent and non-adherent cells were collected and washed in 1x PBS, and fixed in 50% ethanol at 4°C overnight. Cells were then stained with PI solution containing RNase A (Sigma) for 15 minutes at 37°C. DNA content analysis was performed by a FACS caliber cytometer.

### Western blot analysis

Cells (1×10^5^) were typsinized, washed with 1x PBS, and resuspended in 100ul RIPA buffer (Sigma) supplemented with protease inhibitor cocktail then incubated on ice for 40 minutes. After centrifugation, the protein concentration was quantified using Bradford Protein Assay Kit (Bio-Rad). Fifty micrograms (50μg) of total cell lysates were separated using 4-15% SDS-PAGE gradient gels (Bio-Rad) and then transferred to nitrocellulose membranes. Nitrocellulose membranes (Bio-Rad) were blocked using 5% non-fat milk for 1 hour at room temperature, and then incubated with indicated primary antibodies for overnight. Anti-rabbit horseradish peroxidase-conjugated secondary antibody (Bio-Rad) was used. Immunoblots were developed by enhanced chemiluminescence (PerkinElmer).

### Assessment of apoptosis

One million (1×10^5^) PC-3 and PC3-AR cells were seeded in 6-well plates overnight to adhere. Cells were treated with PAN or BEZ235 for 24 and 48 hours. After treatment, cells were collected, washed with 1x PBS, and incubated with annexin:1x annexin binding buffer (1:100) (BD Bioscience) for 10 minutes at room temperature. Annexin positive cells were detected using a FACS caliber cytometer.

### Immunohistochemistry

Mice were sacrificed by CO_2_ asphyxiation at defined time points. Tumor tissue was fixed in 10% buffered formalin overnight followed by an additional 24 hours in 70% ethanol. For antigen retrieval, slides were boiled for 10 minutes in 10mM sodium citrate pH 6 solution for all antibodies. ImmPRESSTM detection system (Vector Laboratories) was used for detection of all primary antibodies. Staining was visualized using 3,3′-Diaminobenzidine (DAB) (Sigma, Saint Louis, MO, FAST 3,3′-Diamino benzidine) and slides were counterstained with hematoxylin. For quantitation of IHC staining representative images (3-6) were obtained using a Zeiss light microscope (Zeiss). Positive nuclear staining was quantified by Aperio ImageScope (v11.1.2.760).

### *In vivo* animal studies

The Institute Animal Care and Use Committee at Roswell Park Cancer Institute approved all mouse protocols used in this study. Mice were housed in an animal facility maintained on a 12-h light/dark cycle, at a constant temperature (22±2°C) and relative humidity (55±15%). Tap water and food were available *ad libitum*. Five million (5×106) PC3 or PC3-AR cells were subcutaneously injected into intact male SCID mice. Treatment was initiated when tumor size reached ~50mm2, mice were randomized to four treatment groups: (1) Vehicle (*N*-Methyl-2-pyrrolidone (NMP)/PEG300, 1:9 v/v: Mon-Fri, oral gavage), (2) BEZ235 25mg/kg (Mon-Fri, oral gavage), (3) PAN 10mg/kg (Mon-Wed-Fri, i.p.), (4) combination. Mice were weighed weekly to monitor for toxicity and tumor growth was assessed by serial caliper measurements twice weekly.

### Statistical Analysis

Data are displayed as the mean ± SE. Differences were determined using one or two-way ANOVA and two-tailed paired t tests where indicated, using GraphPad Prism software. P values less than 0.05 were assigned statistically significant.

## Supplementary Tables and Figure


