# Supplementary material for: Combinatorial antitumor effect of HDACs and the PI3K-Akt-mTOR pathway inhibition in a Pten deficient model of prostate cancer
Source: Oncotarget. 2013 Sep 27;4(12):2225–36. doi: 10.18632/oncotarget.1314 (PMC3926822; doi:10.18632/oncotarget.1314)
Supplement: Supplementary file 1 [file oncotarget-04-2225-s001.pdf]

Combinatorial antitumor effect of HDACs and the PI3K-Akt-mTOR pathway inhibition in a PTEN deficient model of prostate cancer – Ellis et al

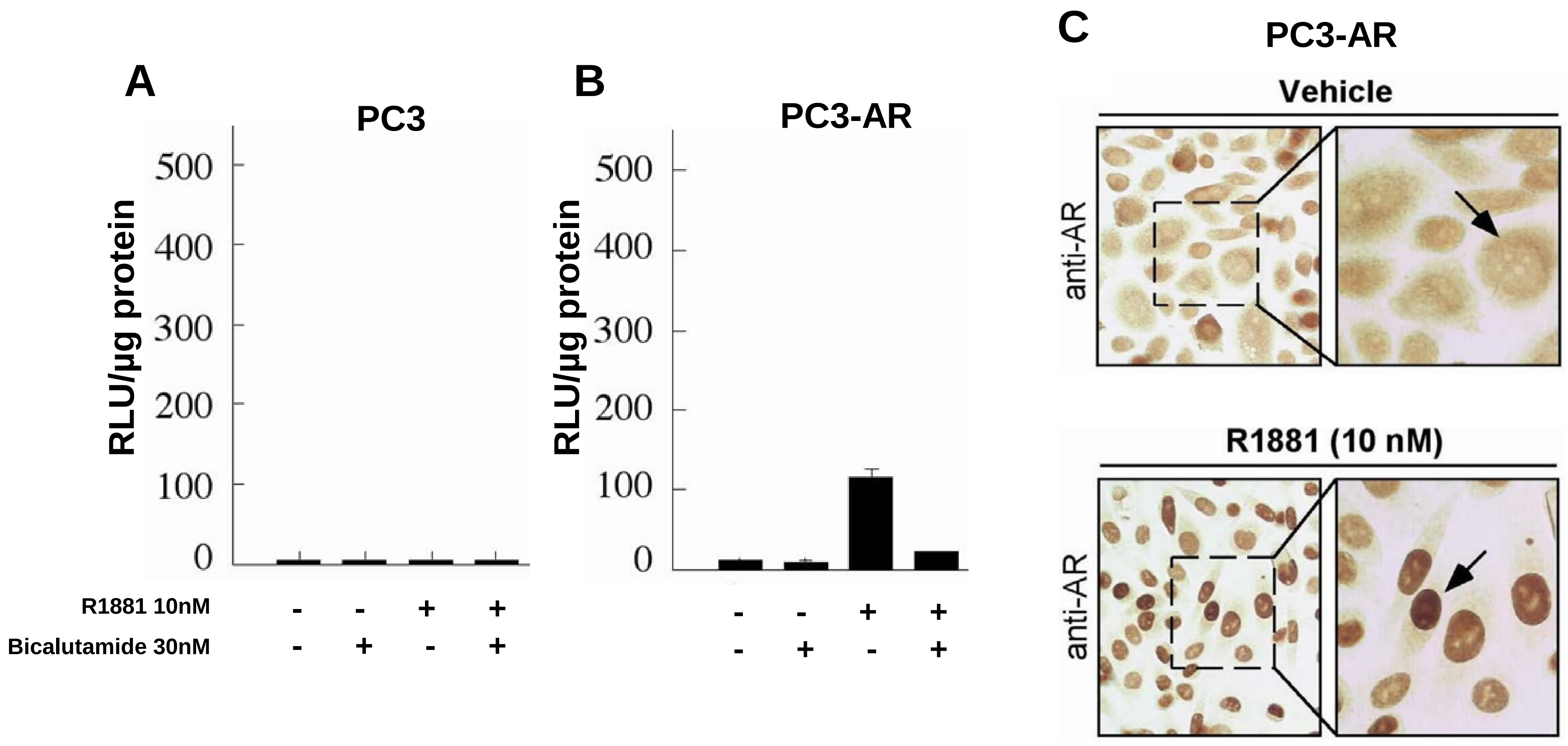

Supplement Fig. 1: (A) PC3 and PC3-AR cells grown in 24-well plates were washed, and incubated for 12 h in RPMI 1640, DMEM, or endothelial cell growth media, respectively, containing 5% (vol/vol) charcoal-stripped FBS. Cells were infected for 3 h with an adenoviral expression vector encoding an MMTV promoter-driven luciferase reporter (multiplicity of infection between 10 and 20 ifu/cell). After infection, cells were incubated 36 h in the corresponding media containing 5% (vol/vol) charcoal-stripped FBS and supplemented with 10nM R1881. For demonstration of inhibition of MMTV-driven luciferase reporter activity by the antiandrogen bicalutamide. At the conclusion of the incubation, cells were washed with PBS and lysed in 50 l reporter lysis buffer (Promega, Madison, WI), followed by a freeze-and-thaw incubation to ensure lysis. Lysates (25 l) were assayed for luciferase activity using the Luciferase Assay System (Promega) and Veritas microplate luminometer (Turner BioSystems, Sunnyvale, CA). Luciferase values were normalized to total protein content measured using the Bradford assay (Bio-Rad Laboratories). All experiments were performed in triplicate **(B)** PC3/AR^{wt} cells were incubated in androgen depleted cell culture conditions for 24hrs. PC3/AR^{wt} were treated with vehicle (left panel) or 10nM R1881 (right panel) for a further 24hrs. Cells were fixed *in situ* using 4% (wt/vol) paraformaldehyde for 30 min at room temperature. Endogenous peroxidase activity was inhibited with 0.3% (vol/vol) H_2O_2 in methanol, and non-specific binding to antibodies was blocked with 3% (wt/vol) BSA for 30 min at room temperature. Cells were incubated overnight with rabbit anti-AR (N-20) antibody (1:100; Santa Cruz Biotechnology). All antibodies were diluted in 100 mm Tris-HCl buffer (pH 7.8) that contained 8.4 mM sodium phosphate, 3.5mm potassium phosphate, 120mm NaCl, and 1% (wt/vol) BSA. After washing three times in Tris-HCl buffer (pH 7.8) for 10 min each, specimens were incubated with horseradish peroxidase (HRP)-conjugated antirabbit IgG (1:100; Dako) for 2hrs at room temperature. Peroxidase activity was developed using 100 mM Tris-HCl buffer that contained 3,3 diaminobenzidine tetrahydrochloride (1µg/ml; Sigma-Aldrich, St. Louis, MO) and H_2O_2 (1µl/ml; VWR Intl., West Chester, PA). Stained slides were dehydrated by sequential steps through a graded series of alcohol washes and Citrisolv (Fisher Scientific International, Suwannee, GA), and mounted using coverslips.

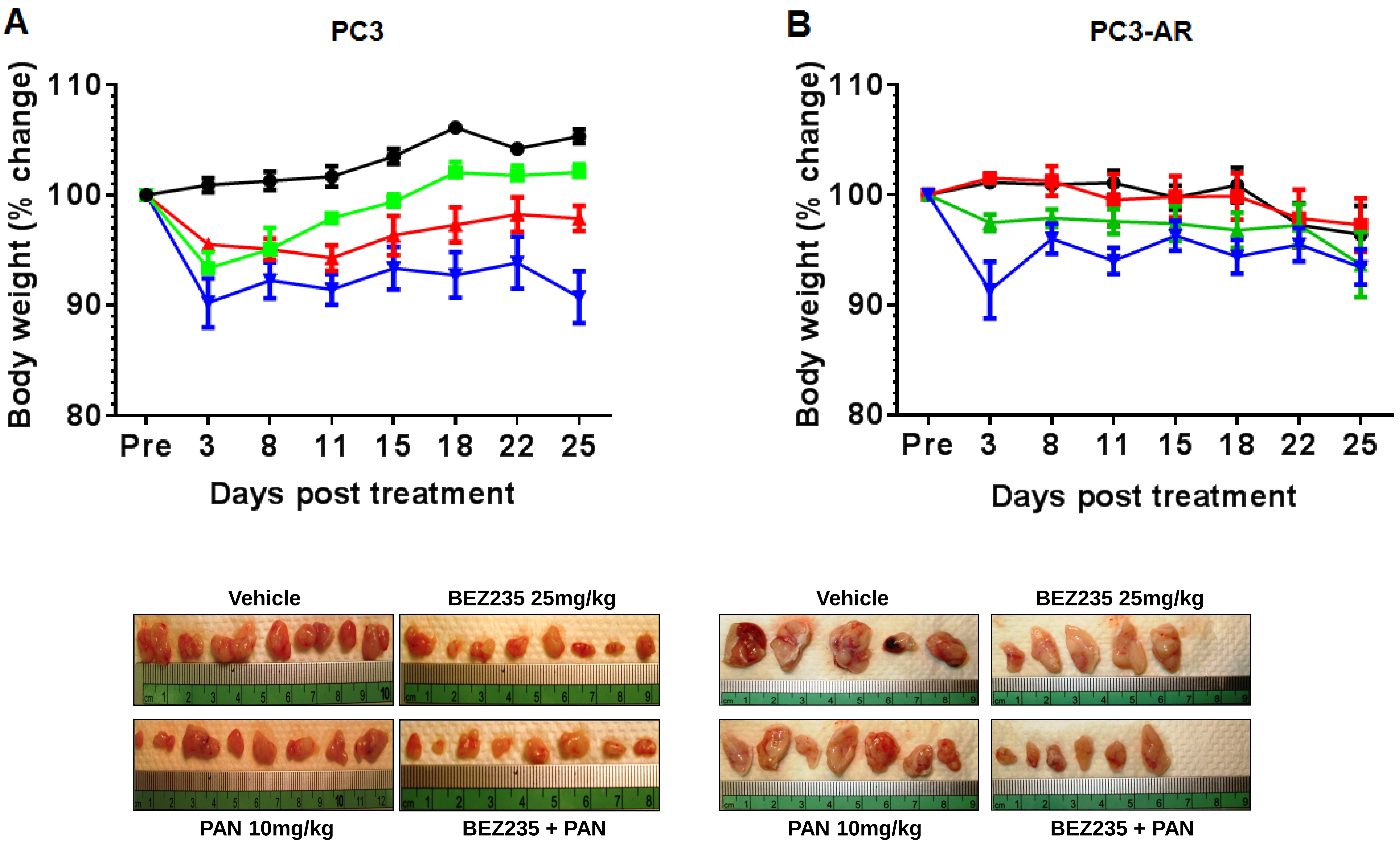

Supplement Fig. 2: (A) PC3 or **(B)** PC3-AR cells ($5x10^6$) were injected subcutaneously in the flank of intact SCID male mice. Treatment was initiated when tumors measured approximately $50mm^2$ (L x W). Mice were treated with vehicle (NMP:PEG300 1:9, 5d on 2d off, oral gavage, PC3 *n=8*, PC3-AR *n=5*), BEZ235 (25mg/kg, 5d on 2d off, oral gavage x5 weekly, PC3 *n=8*, PC3-AR *n=6*), panobinostat (PAN) (10mg/kg, Mon-Wed-Fri, i.p., PC3 *n=8*, PC3-AR *n=7*), or combination (PC3 *n=8*, PC3-AR *n=6*). Toxicitiy was monitored by weighing mice biweekly. Each treatment group was normalized to the pretreatment measurements and converted to percent tumor growth. Each point represents mean tumor size ±SE. For PC3 tumors. (Bottom panels): photographs of all PC3 and PC3-AR tumors at conclusion of therapy. Vehicle (black line), BEZ (green line), PAN (red line), Combination (blue line).

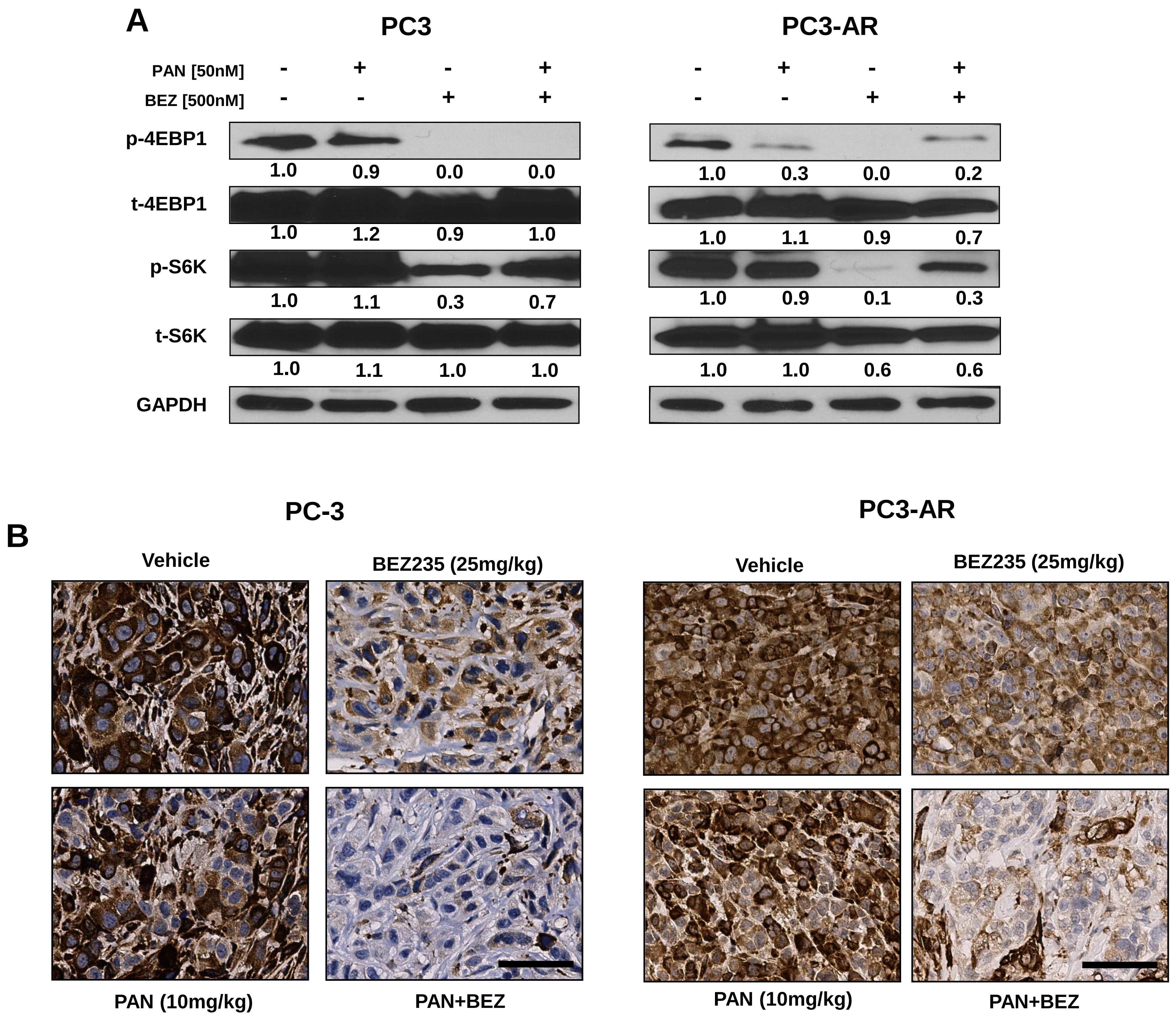

Supplement Fig. 3: (A) PC3 and PC3-AR cells were treated with indicated concentrations of panobinostat (PAN) and/or BEZ235 for 24 hours. Following treatment, total cell lysates were prepared and immunoblot analysis was performed for p-4EBP1 (cell signaling), total 4EBP1 (cell signaling), p-S6K (cell signaling) and total S6K (cell signaling). The expression of GAPDH in lysates served as loading control. **(B)** Example photomicrographs of p-S6K immunostained PC3 and PC3-AR tumor tissue.
